# A neuron-in-capillary platform for facile collection and mass spectrometric characterization of a secreted neuropeptide

**DOI:** 10.1038/srep26940

**Published:** 2016-06-01

**Authors:** Chang Young Lee, Yi Fan, Stanislav S. Rubakhin, Sook Yoon, Jonathan V. Sweedler

**Affiliations:** 1 Department of Chemistry and the Beckman Institute, University of Illinois, Urbana, IL 61801, USA; 2 School of Energy and Chemical Engineering, School of Life Sciences, Ulsan National Institute of Science and Technology (UNIST), Ulsan 44919, Republic of Korea

## Abstract

The integration of microfluidic devices—which efficiently handle small liquid volumes—with separations/mass spectrometry (MS) is an effective approach for profiling the neurochemistry occurring in selected neurons. Interfacing the microfluidic cell culture to the mass spectrometer is challenging because of geometric and scaling issues. Here we demonstrate the hyphenation of a neuron-in-capillary platform to a solid phase extraction device and off-line MS. A primary neuronal culture of *Aplysia californica* neurons was established directly inside a cylindrical polyimide capillary. The approach also uses a particle-embedded monolith to condition neuropeptide releasates collected from several *Aplysia* neurons cultured in the capillary, with the subsequent characterization of released peptides via MS. This system presents a number of advances compared to more traditional microfluidic devices fabricated with polydimethylsiloxane. These include low cost, easy access to cell culture, rigidity, ease of transport, and minimal fluid handling. The cylindrical geometry of the platform allows convenient interface with a wide range of analytical tools that utilize capillary columns.

Neuropeptides are important cell to cell signalling molecules in the nervous system that are involved in a wide range of functions such as tactile sensations, food intake, reproduction, learning, and memory^1^,^2^,^3^. They are synthesized and stored within neurons and released into the extracellular space where they then act as neurotransmitters, neuromodulators, and hormones. Extracellular fluids collected *in vivo* from a brain region typically contain chemical information from a large number of cells^4^. Therefore, *in vitro* analysis of released neuropeptides from sparse neuronal networks or even individual neurons helps one to decipher the complex cell to cell chemical signalling associated with mechanisms of physiological processes, which are difficult to study when using larger *in vivo* ensemble measurements.

Mass spectrometry (MS) is an effective approach for the identification and quantitation of neuropeptides because of its excellent detection limits and high information content. Incorporating MS detection with a microfluidic system adds chemical information to cellular analyses but requires careful design of the interface, especially when working with low numbers of cells; in such cases, it is important to minimize sample loss and cross-contamination^5^. With appropriate surface treatment and carefully designed fluidics, an integrated microfluidic system is capable of analysing neuropeptide releasates from living neurons. Previous examples of successful microfluidics–MS integration include detection of glutamate released from live PC12 cells^6^, analysis of droplet-in-oil microdialysates collected *in vivo* from the rat brain^7^, detection of angiotensin^8^ or secreted insulin^9^ by matrix-assisted laser desorption ionization (MALDI) MS in droplet-in-oil collection experiments, and *ex situ* MALDI MS imaging of neuropeptide releasates deposited onto a C18-treated substrate^10^,^11^.

The sea slug *Aplysia californica* has become an effective animal model for investigating single neuron biochemistry and physiology because its large, well-characterized cells form relatively simple neuronal networks. Due to its importance in single cell studies, the culture of individual *Aplysia* neurons in microchannels made with the elastomer polydimethylsiloxane (PDMS) has been established in our laboratory^12^. Maintaining a robust low-density neuronal culture within a microfluidic device that enables efficient collection of peptide release is a challenge when the system only involves a few cells. Many microfluidic devices are made of PDMS; the material has advantages in terms of its optical transparency and oxygen permeability, but may exhibit cytotoxicity due to residual solvents, catalysts, and un-crosslinked oligomers, and it may absorb peptides. Although this material may not reduce cell viability for *Aplysia* bag cell neurons, low-density culture with mammalian neurons has been challenging in PDMS microchannels. By removing cytotoxic factors via extensive chemical treatment, Millet *et al*.^13^ was able to establish cultures of individual hippocampal neurons in a PDMS device. However, the treatment requires several days of frequent solvent exchange, and peptide absorption still occurs.

The hyphenation of MS to microfluidic devices presents another challenge. For instance, efforts have been made to better interface microfluidics with electrospray ionization (ESI) MS by generating the electrospray (1) directly from the edge of a microfluidic chip, (2) from an ESI needle positioned inside the device, or (3) from an integrated microfabricated emitter^5^. Here we have chosen to work with offline MALDI MS because it separates the requirements of cell culture from the analyte characterization process, and has been used to characterize secretion from larger numbers of cells^14^. The majority of microfluidic devices have channels with rectangular cross-sections, whereas capillary columns used in analytical techniques such as liquid chromatography or capillary electrophoresis have a circular form. Improvement in the performance of microfluidics–MS systems may be achieved by choosing an optimal geometry interface, which often relates to analyte band-broadening and dilution of analytes^15^,^16^. Hence, using a simple tubular device for cell culture and extracellular environment manipulation that has similar geometry with a multitude of sample conditioning and manipulation devices may be a promising approach for the online and offline hyphenation of microfluidics to MS.

The goal of this work was to design a small-volume capillary device for culturing neurons that would allow us to apply secretagogues to the cells, and collect, condition and characterize their neuropeptide releasates. Here we measured the release from a limited number of primary *Aplysia* neurons cultured within an untreated polyimide capillary. This neuron-in-capillary system can be readily removed from the culture medium while still maintaining cell viability. Furthermore, unlike the typical PDMS-based rectangular platforms, the cylindrical geometry of the system allows for direct connection to a capillary-based particle-embedded monolithic capillary (PEMC)^17^ for the collection step, followed by MALDI-time-of-flight (TOF) MS analysis of neuropeptides released from as few as two neurons.

## Results and Discussion

### Neuronal culture inside a polyimide capillary

**Figure 1:**
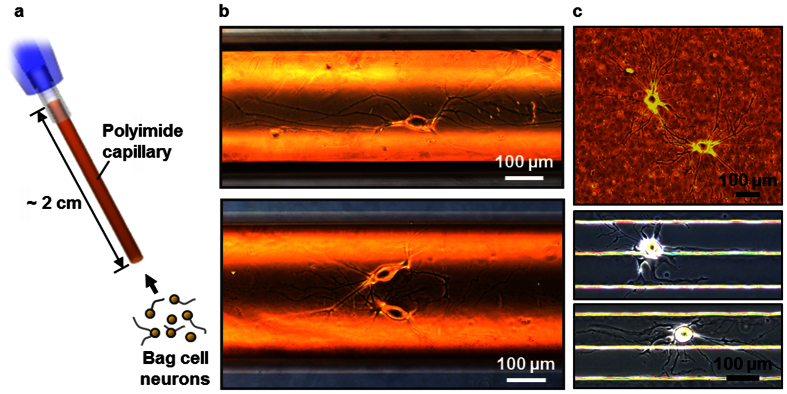
The neuron-in-capillary platform. (**a**) Schematic of the neuron-in-capillary platform where a polyimide capillary is attached to small-volume flexible polymer tubing. Liquid flow allows loading of isolated neurons into the polyimide capillary. (**b**) Individual (top, 5 days *in vitro* (DIV)) and network-forming (bottom, 3 DIV) bag cell neurons of *Aplysia* in a bare polyimide capillary. (**c**) Bag cell neurons cultured on other untreated polyimide substrates: Kapton® polyimide tape (3 DIV, top), and line/space patterns of thermally imidized polyamic acid (7 DIV, bottom).

Figure 1a illustrates the loading of neurons into a polyimide capillary, prepared by etching the inner silica of the capillary with hydrofluoric acid. Bag cell neurons, which are located in two clusters in the abdominal ganglia of an adult *Aplysia*, were selected as the model system; each cluster is comprised of about 400 bag cell neurons^18^. These cells are robust in culture, biochemically well characterized^10^,^19^,^20^, and therefore well suited for evaluating our new analytical platform. With a custom-made micropipette attached to one end, isolated bag cell neurons were loaded into the capillary by gentle suction. For incubation, the neuron-in-capillary system was immersed in artificial sea water (ASW, pH 7.8) at 14 ^o^C. Figure 1b shows individual (top, 5 days *in vitro* or DIV) and network-forming (bottom, 3 DIV) bag cell neurons cultured inside the capillary. In terms of the length and branch structures of the neurites, these cells were comparable to, or often more proliferative, than previously reported dish cultures. A cell viability of over 80% can be routinely achieved throughout any axial location of the capillary.

A distinctive feature of our neuron-in-capillary system is that the neurite outgrowth is preferential along the axial direction of the capillary, the direction of zero curvature (Fig. 1b). Curvature (*K*) of the interior of a cylinder is a function of radius (*r*) and the angle relative to the axis (*θ*)^21^.


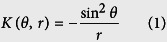


Cells inside the capillary experienced a negative curvature of −3.1 × 10^−3 ^μm^−1^ along the circumference (*r* = 320 μm, *θ* = 90^o^). Even with a capillary diameter of about 6-fold larger than the soma, we observed the orientation of neurites along the capillary axis, and this phenomenon was more pronounced for longer neurites. Smeal *et al*.^21^,^22^ also observed the axial orientation of dorsal root ganglia neurons as the size of soma became close to the filament diameter, but the culture in their study was on the exterior of a polypropylene filament. As there are no adhesion-promoting molecules in our system, the neurite guidance, if any, was not caused by the orientation of those molecules along the curved surface. Hence, we conclude that the effect is mechanical, and the results may be related to bending-induced mechanical strain on the cytoskeleton of the neuron^21^.

It should be noted that the cells attach and grow on the surface of the polyimide capillary without surface treatments. We found this to be true for several types of polyimide substrates, including commercially available Kapton® tape (DuPont) attached to a glass coverslip (Fig. 1c, top), a film of partially imidized polyamic acids (data not shown), and a patterned polyimide that exhibited neurite guidance properties (Fig. 1c, bottom). The cell viability test using fluorescein diacetate confirms that the cells are viable when cultured on untreated polyimide substrates (Supplementary Fig. S1). The culture of adhering cells *in vitro* usually requires substrates treated with cell adhesion promoters such as poly-D/L-lysine, collagen, fibronectin, etc^23^. The quality and reproducibility of these coatings depends on many parameters, including the chemical purity and stability of the compounds and their layers. A polyimide substrate, in contrast, is repeatable and stable. Culturing cells directly on the substrate without surface treatment reduces the time and effort needed for sample preparation and enhances the ability to prepare reproducible culturing surfaces. Polyimide is flexible, nonconductive, water-impermeable, resistant to heat, and chemically inert. The material has been widely used in electronics and has found applications in biology and chemistry. Examples include substrates for cell culture^24^,^25^,^26^, structures for cell guidance^27^,^28^, microfluidic channels with embedded electrodes^29^, microfluidic liquid chromatographic separation for peptide analysis^30^, and tissue implants^31^,^32^ or encapsulants^33^ for implantable medical devices. Therefore, our findings are in line with previously observed properties of the material. We found that the cell culture supporting capabilities of untreated polyimide is true for both bag cells and cell lines such as HEK293T cells (Supplementary Fig. S2). The ability of cylindrical polyimide devices to support invertebrate neuronal growth and network formation, as well as mammalian cell culture, expands the application of polyimide-based devices in cell biology and neuroscience.

There have been a limited number of reports on the design and application of cell-in-capillary systems, although it appears none have been used for the analysis of cell to cell chemical signalling. For instance, a cell-in-capillary system was shown to have potential in clinical oncology for drug sensitivity testing^34^, with the system used to select effective chemotherapeutic agents. Forming a pure colony from a patient’s tumour cell, although necessary for selecting chemotherapeutic agents most effective to the patient, has been challenging using conventional two-layer agar Petri dishes^34^. The ability to culture a single isolated cell and its subsequent proliferation may be a promising alternative. However, efforts to do so had been unsuccessful until 1948 when Sanford *et al*.^35^ used a glass capillary as a culturing vessel. The key was in restricting the volume so that the originally inadequate medium could be self-adjusted by the cell for its survival. The low cross-sectional width and high aspect ratio of their vessel minimized the pH change as well as the evaporation of extracellular media. When applied to non-dividing cell types and using a smaller diameter capillary, this type of platform is well-suited for studying single cells, e.g., the analysis of neuropeptides from individual neurons.

### Integration with a PEMC column to condition and characterize released neuropeptides via MS

Capillary-based cell culture has several advantages as a platform for the chemical analysis of secretions from living cells. First, the culture is robust because the capillary enclosure reduces the effect of shear force, which develops in an open dish culture medium with dish movements. Uniformly high cell viability throughout the axial capillary suggests a sufficient diffusion-related reduction of metabolic wastes and cytotoxic compounds within the capillary (~320 μm ×2 cm, volume ~1.6 μL). Cells attached at all radial locations can be visualized using a microscope. Furthermore, the surface tension of water holds the cell culture media within the capillary, even when the capillary is taken out of the culture bath and tilted. The amount of evaporation of the media while the capillary ends are in the air is insignificant for a duration of minutes. Therefore, the neuron-in-capillary can be handled in air without degrading the cell viability, which allows convenient interface with other analytical platforms.

**Figure 2:**
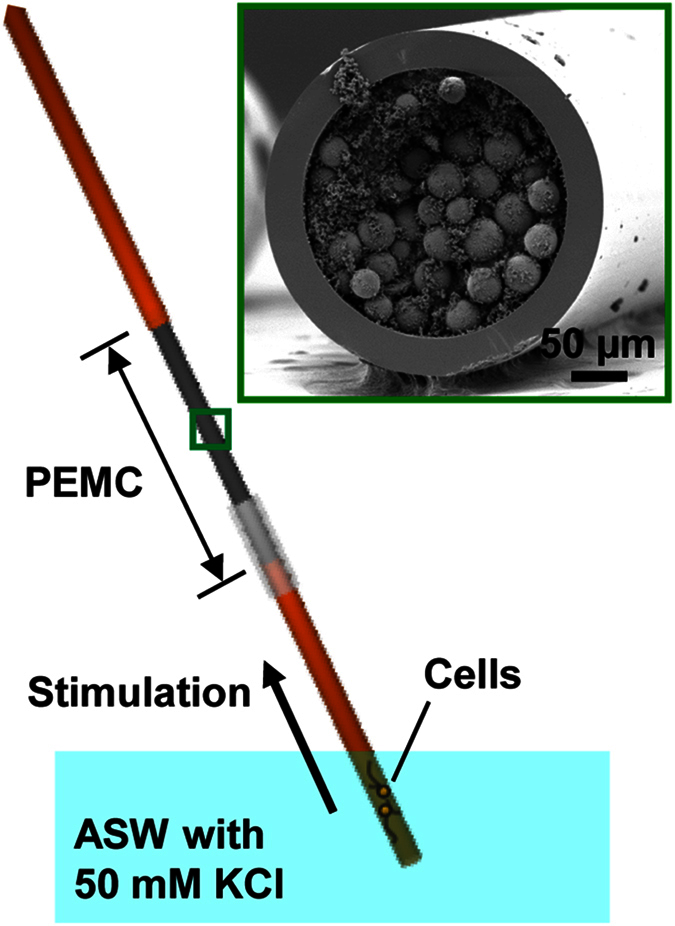
Integration of the neuron-in-capillary with a PEMC. Neuropeptides released from the stimulated bag cell neurons are collected by the PEMC. Inset shows a cross-sectional view of the PEMC obtained using scanning electron microscopy.

The tubular geometry, along with the aforementioned advantages, is distinct from the typical rectangular PDMS-based microfluidic systems and enables facile connection with capillary-based or interfaced analytical platforms such as LC-ESI-MS. We demonstrate this by connecting the neuron-in-capillary to a PEMC column for subsequent neuropeptide assay (Fig. 2). We have previously demonstrated the mass spectrometric analysis of neuropeptide release from a single *Aplysia* neuron using a PEMC^17^. The inset in Fig. 2 is a cross-sectional electron microscope image of the packed capillary, which has a large surface area that efficiently traps neuropeptides. While the column was linked to the PEMC, it was connected to a syringe to generate negative pressure. The cell-containing end of the neuron-in-capillary platform was immersed into a 50 mM KCl in ASW solution and the syringe pulled the solution into the assembly, thereby causing the depolarization of the cell membrane and subsequent release of neuropeptides. The released peptides were retained on the PEMC column. Next, the neuron-in-capillary platform was disconnected from the PEMC column and returned to the culture medium. The platform would potentially allow multiple cycles of cell incubation and stimulation. The peptides collected on the PEMC were then eluted and deposited onto a MALDI MS target for MS-based characterization.

**Figure 3:**
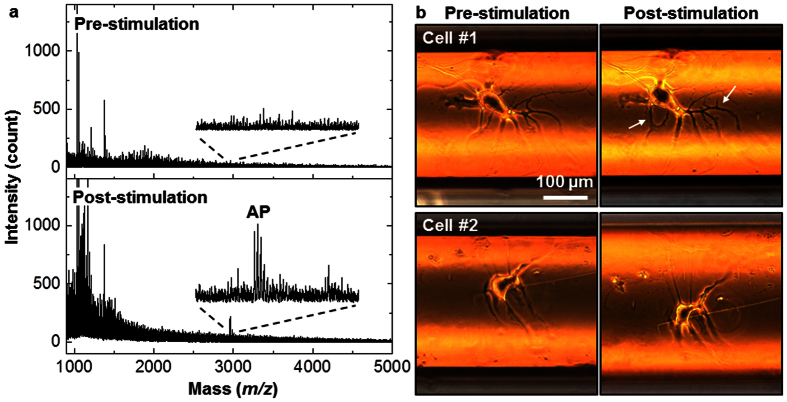
Mass spectrometric detection of released neuropeptides from two bag cell neurons cultured in a capillary. (**a**) Mass spectra of neuropeptide releasates collected by the PEMC. While no peptides were detected at pre-stimulation (top), acidic peptide (AP, *m/z* 2959.6) was detected after stimulation with KCl. (**b**) Cells exposed to elevated extracellular KCl content retain their morphology, which suggests cellular integrity and the absence of intracellular analyte release due to cell lysis. Microphotographs of two representative cells before and after KCl stimulation. Arrows in the Cell #1 microphotograph indicate neuronal processes that are further extended upon stimulation.

Using the neuron-in-capillary platform integrated with a PEMC column, we collected neuronal releasates from two cultured bag cell neurons of *Aplysia californica*. We then used MALDI MS to characterize any neuropeptides from the release (Fig. 3a). Acidic peptide (AP, *m/z* 2959.6) was detected in the samples collected during the cell stimulation, but not in the samples acquired before the application of the elevated potassium. The variation of signal intensity with the position of the neurons in the capillary was insignificant compared to the intrinsic variation of the MALDI MS signal. According to our previously published quantitative analysis^11^, a single bag cell neuron stimulated by KCl releases 0.15 ± 0.03 pmol of AP and 0.13 ± 0.06 pmol of α-bag cell peptide (BCP, m/z 1122.6). Hence, the amount of AP detected in this system corresponds to about 300 fmol. For quantitation of the peptides the neuron-in-capillary platform can be connected to the microfluidic device devised by us^11^, and the amount of peptide released measured by the length-to-amount conversion approach. This demonstrates the utility of the hyphenated platform to study cell to cell signalling. However, we did not detect egg-laying hormone (ELH, *m/z* 4382.4), which was also expected to be released; ELH and AP are from the N-terminus of egg laying prohormone and are packaged into the same vesicles^36^ and should have been co-released. The lack of an ELH signal may be due to its higher molecular mass, which lowers its detectability. α-, β-, and γ-BCP, which we did not detect either, are packaged together but into a distinct vesicle class from the vesicles containing AP and ELH. The reason why none of these C-terminal peptides were detected is not clear and requires further study. The amounts of α-BCP and ELH expected to be released, but not detected here, are probably around 0.26 pmol and 0.32 pmol, respectively^11^,^36^. We do not believe that the absence of ELH and BCP was due to the polyimide capillary, and we tested this by placing the cells near the end of the capillary close to the PEMC; in this case, we still detected AP only.

When studying neuropeptide release from a limited number of cells, it is important to verify that analyte signals represent physiologically released molecules but not compounds leaked from damaged cells. The viability of cells during experiments can be affected by a number of factors, including mechanical forces created during the connection of the PEMC column, cell stimulation with KCl, and disconnection of the neuron-in-capillary platform from the PEMC column. Every stimulated neuron was examined visually under a microscope before assembling the system and after its disassembly. Figure 3b shows two different cells (#1 and #2) at the pre-stimulation stage (left) and post-stimulation stage (right). The intact morphology of the cell bodies and neurites support the assessment that the neurons weathered the manipulations well. Our observations, and the results of others, indicate that when *Aplysia* neurons are damaged, they exhibit easily visualized blebbing on the surface of the cell body and a change in the morphology of their terminals, features we did not observe.

Surprisingly, we observed that after the KCl stimulation, some neurites exhibited additional outgrowth (Fig. 3b, cell #1). The stimulation/release collection experiment takes about 40 min (20 min each for pre-stimulation and stimulation phase); therefore, the neurites extended within this relatively short time period. Furthermore, in some cases, cells without any visible neurites at 1 DIV also started extending neurites when stimulated by KCl. However, this study did not focus on differentiating the mechanical influences of liquid flow versus KCl-assisted cell stimulation. Although we did not examine this issue, the impact of mechanical forces on bag cell neurons has been shown to change outgrowth and vesicle clustering when using a PDMS platform^37^. Such behaviours based on flow are worth exploring in the future to understand the growth and (re) generation of neuronal networks.

**Figure 4:**
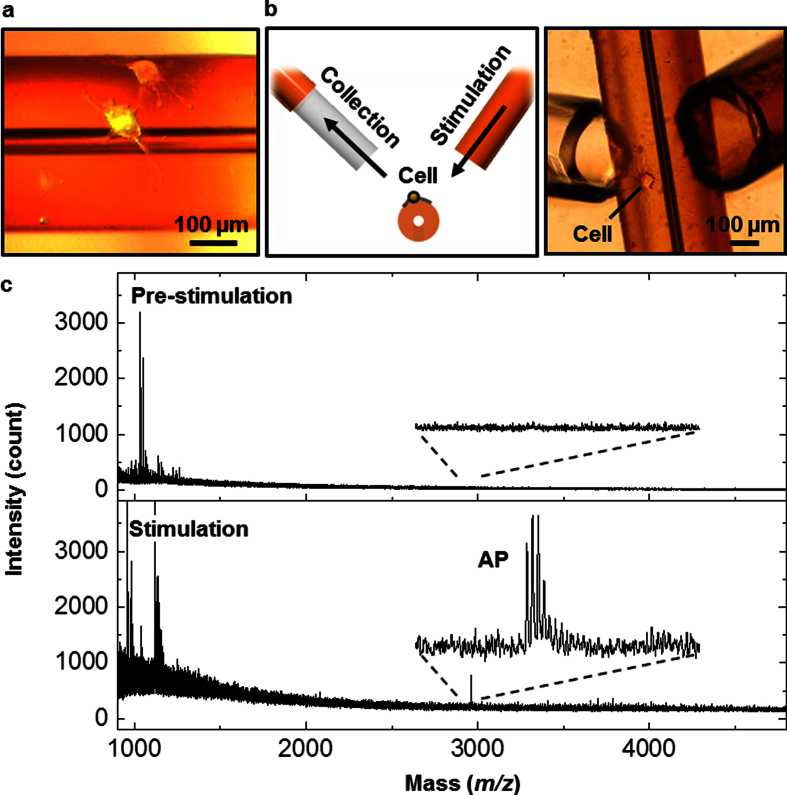
Collection and analysis of neuropeptides from neurons cultured on the outside surface of a polyimide capillary. (**a**) Microphotograph of two neurons with developed processes on top of the polyimide capillary. (**b**) Schematic of release stimulation and collection in which a high content KCl solution is applied through the stimulation capillary, and releasates are drawn into the collection capillary. (**c**) Mass spectra of neuropeptide releasates from a single bag cell neuron collected by the PEMC device assembled in line with the collection capillary. While no peptides were detected at pre-stimulation (top), acidic peptide (AP, *m/z* 2959.6) was detected after stimulation with KCl.

Further questions arise about whether the curved substrate affects neuropeptide release. We repeated the measurement with *Aplysia* bag cell neurons cultured on the convex exterior of the polyimide capillary (Fig. 4a). For this experiment, etching out the inner silica was not necessary. As a direct connection to a PEMC is not as straightforward in this case, we took a dual-capillary approach^17^ devised previously in our laboratory. Briefly, two capillaries were placed close to the cells, one being a standard fused-silica column and the second a PEMC, as shown in Fig. 4b. Cells were then stimulated by flowing ASW with elevated KCl through the former, while the releasates were collected by the latter. Figure 4c shows mass spectra of the releasates from a single bag cell neuron before (top) and after (bottom) the stimulation. The results are similar to what was observed from the concave interior of the capillary. We did not repeat the test on a planar polyimide substrate, but the results above suggest that the neuropeptide release pattern of *Aplysia* bag cell neurons is insensitive to the curvature of the substrate.

## Conclusions

The neuron-in-capillary platform presented here is robust and easily integrated with capillary-based analytical tools. The addition of a PEMC for the mass spectrometric characterization of neuropeptide releasates allows for monitoring the chemical signalling between live neurons, and potentially enables multiple cycles of release–collection–analysis. In principle, this platform can be hyphenated to existing analytical systems, including liquid chromatography and ESI MS, via column fittings and automated valves. Quantitation of neuropeptide releasates should be possible by connecting the neuron-in-capillary platform to another microfluidic system that relates the length of the C18 surface retaining analyte to the amount of retained peptide^11^. Hence, our approach will find broad applications in the analysis of mass-limited biological samples. Furthermore, when combined with microfabrication techniques, successful neuronal culture on a bare polyimide substrate opens up new opportunities for application in neuroscience and engineering investigations. Examples include studying neurons in a 3D polyimide structure that mimics the cellular microenvironment, a neuronal network on a polyimide substrate with built-in microelectronic circuitry, and a polyimide microstructure for nerve guidance. We have demonstrated, using HEK293T cells, that cell culture in the capillary can be developed not only for invertebrate cells but also for mammalian cell types. The utility of the capillary platform can be further explored in different functional studies utilizing individual cells and networks of mammalian neurons and other types of *Aplysia* neurons.

## Methods

### Reagents

Artificial seawater (ASW) contained 460 mM NaCl, 26 mM MgSO_4_, 22 mM MgCl_2_, 15 mM HEPES, 10 mM KCl, 10 mM CaCl_2_, and 2.5 mM NaHCO_3_, all purchased from Sigma-Aldrich (St. Louis, MO). The solution was adjusted to pH 7.8 and supplemented with antibiotics. Methanol, acetonitrile (ACN), formic acid (FA), and ethylene glycol dimethacrylate 98% (EDMA) were purchased from Fisher Scientific (Fairlawn, NJ). Stearyl methacrylate (SMA), 3-(trimethoxysilyl)propyl methacrylate 98% (TMSPM), iso-amyl alcohol (IAA), 2,2′-azobis(2-methylpropionitrile) (AIBN), and 1,4-butanediol (BD) were obtained from Sigma-Aldrich.

### Polyimide capillary and cell loading

Polyimide capillaries, about 2-cm-long, were prepared from polyimide-coated silica capillaries (TSP050375, 360 μm outer diameter (o.d.), 50 μm inner diameter (i.d.), 18 μm-thick polyimide coating, Polymicro Technologies, Phoenix, AZ) (Fig. 1) by etching the core silica with 49% hydrofluoric acid. Tubing was attached at the end of each capillary to serve as a connector to a micropipette for loading cells. Right before loading the cells, the capillary was connected to a micropipette, which was custom-made from silicone tubing, and a 10 μL pipette tip. Cells were loaded into the capillary by gently compressing and releasing the micropipette while counting the number of cells under a stereozoom microscope (Leica, MZ75, Buffalo Grove, IL). When the desired number of cells were loaded, the capillary was separated from the micropipette by carefully cutting the connector tubing with a razor blade, thereby preventing cells from being unloaded from the capillary. The capillaries were then incubated at 14 ^o^C for 1–3 days until the release experiment was performed.

### Cell culture on various polyimide substrates

Kapton® tape (Dupont, Wilmington, DE) was cut and attached on a glass coverslip, and used immediately in ASW for cell culture. Poly(pyromellitic dianhydride-co-4,4′-oxydianiline)amic acid solution was purchased from Sigma-Aldrich. The solution was spin-coated on a glass coverslip at 3000 rpm for 30 s, followed by partial curing at 110 ^o^C for 2 min on a hotplate. The line/space patterns were created by photolithography and reactive ion etching under an O_2_ flow rate of 20 sccm, pressure of 50 mTorr, power at 150 W, and etching time of 7 min.

### Animal dissection

An adult *Aplysia californica* (~100 g) was anesthetized by injecting a half body weight of isotonic MgCl_2_ solution (0.39 M). The abdominal ganglion was dissected and treated with protease XIV from *Streptomyces griseus* (Sigma-Aldrich) (10 mg/mL in ASW) at 34 ^o^C for 90 min. Bag cells were then manually isolated from the ganglion and loaded into polyimide capillary as described above.

### Fabrication of the PEMC columns

The PEMC columns were prepared as reported previously by our laboratory^17^. Briefly, fused silica capillaries (250 μm i.d., 360 μm o.d., Polymicro Technologies), rinsed with aqueous solutions containing NaOH and HCl, were filled with a methanolic solution of TMSPM (50%) and incubated overnight at 40 ^o^C. A UV-transparent window was generated by removing the polyimide coating at one end of the capillary. A prepolymerization mixture was prepared by mixing 836 mg IAA, 167 mg BD, 406 mg SMA, and 136 mg EDMA with 1 wt% AIBN and 5 v/v% TMSPM. A slurry was made by adding 20 mg of pyrrolidone particles (Strata-X, Phenomenex, Torrance, CA), with an average diameter of 30 μm, into 100 μL of the prepolymerization mixture. The capillary was filled with the slurry and exposed to UV light (365 nm) for 30 min using a handheld UV lamp (UVP, Upland, CA). The polymerization yields poly(stearyl methacrylate-co-ethylene glycol dimethacrylate), poly(SMA-co-EDMA), which tightly glues the beads together. Residual monomers were removed by thoroughly rinsing the capillary with methanol.

### Extraction and MALDI MS

Before integration with the polyimide capillary, the PEMC column was conditioned with methanol and equilibrated with water. Then the column was connected to the neuron-in-capillary platform for the neuropeptide release–collection experiments described above. Using a syringe pump the neuropeptide releasates from the stimulated cells were passed through the integrated system at 0.25 μL/min. After collection, the PEMC column was detached from the polyimide capillary and rinsed with 5 μL of water to remove the salts, during which less than 5% of peptides were lost as estimated by ^3^H-angiotensin II^17^. The collected neuropeptides were eluted with 3 μL of 2% FA in 90% MeOH. For MALDI-TOF MS analysis, an aliquot (0.25 μL) of the eluted peptides was dropped onto a MALDI target with pre-spotted matrix followed by MALDI MS characterization using an ultrafleXtreme MALDI-TOF/TOF mass spectrometer (Bruker Daltonics, Billerica, MA) with a frequency tripled Nd:YAG solid state laser (λ = 355 nm) as described previously^17^.

## Additional Information

**How to cite this article**: Lee, C. Y. *et al*. A neuron-in-capillary platform for facile collection and mass spectrometric characterization of a secreted neuropeptide. *Sci. Rep*. **6**, 26940; doi: 10.1038/srep26940 (2016).

## Supplementary Material

Supplementary Information

## References

[b1] Gerald C. . A receptor subtype involved in neuropeptide-Y-induced food intake. Nature 382, 168–171 (1996). 8700207 10.1038/382168a0

[b2] Croushore C. A., Supharoek S. A., Lee C. Y., Jakmunee J. & Sweedler J. V. Microfluidic device for the selective chemical stimulation of neurons and characterization of peptide release with mass spectrometry. Anal. Chem. 84, 9446–9452 (2012). 23004687 10.1021/ac302283u PMC3490451

[b3] van den Pol A. N. Neuropeptide transmission in brain circuits. Neuron 76, 98–115 (2012). 23040809 10.1016/j.neuron.2012.09.014 PMC3918222

[b4] Croushore C. A. & Sweedler J. V. Microfluidic systems for studying neurotransmitters and neurotransmission. Lab Chip 13, 1666–1676 (2013). 23474943 10.1039/c3lc41334a PMC3632338

[b5] Gao D., Liu H. X., Jiang Y. Y. & Lin J. M. Recent advances in microfluidics combined with mass spectrometry: technologies and applications. Lab Chip 13, 3309–3322 (2013). 23824006 10.1039/c3lc50449b

[b6] Wei H. B., Li H. F., Gao D. & Lin J. M. Multi-channel microfluidic devices combined with electrospray ionization quadrupole time-of-flight mass spectrometry applied to the monitoring of glutamate release from neuronal cells. Analyst 135, 2043–2050 (2010). 20526497 10.1039/c0an00162g

[b7] Song P., Hershey N. D., Mabrouk O. S., Slaney T. R. & Kennedy R. T. Mass spectrometry “sensor” for *in vivo* acetylcholine monitoring. Anal. Chem. 84, 4659–4664 (2012). 22616788 10.1021/ac301203m PMC3389145

[b8] Kuster S. K. . Interfacing droplet microfluidics with matrix-assisted laser desorption/ionization mass spectrometry: label-free content analysis of single droplets. Anal. Chem. 85, 1285–1289 (2013). 23289755 10.1021/ac3033189

[b9] Chen D. . The chemistrode: A droplet-based microfluidic device for stimulation and recording with high temporal, spatial, and chemical resolution. Proc. Natl. Acad. Sci. USA. 105, 16843–16848 (2008). 18974218 10.1073/pnas.0807916105 PMC2579341

[b10] Jo K. . Mass spectrometric imaging of peptide release from neuronal cells within microfluidic devices. Lab Chip 7, 1454–1460 (2007). 17960271 10.1039/b706940e

[b11] Zhong M., Lee C. Y., Croushore C. A. & Sweedler J. V. Label-free quantitation of peptide release from neurons in a microfluidic device with mass spectrometry imaging. Lab Chip 12, 2037–2045 (2012). 22508372 10.1039/c2lc21085a PMC3558029

[b12] Lee C. Y., Romanova E. V. & Sweedler J. V. Laminar stream of detergents for subcellular neurite damage in a microfluidic device: a simple tool for the study of neuroregeneration. J. Neural Eng. 10 (2013). 10.1088/1741-2560/10/3/036020 PMC3687785 23656702

[b13] Millet L. J., Stewart M. E., Sweedler J. V., Nuzzo R. G. & Gillette M. U. Microfluidic devices for culturing primary mammalian neurons at low densities. Lab Chip 7, 987–994 (2007). 17653340 10.1039/b705266a

[b14] Tillmaand E. G. . Peptidomics and secretomics of the mammalian peripheral sensory-motor system. J. Am. Soc. Mass Spectrom. 26, 2051–2061 (2015). 26392278 10.1007/s13361-015-1256-1 PMC4655166

[b15] Radadia A. D., Morgan R. D., Masel R. I. & Shannon M. A. Partially buried microcolumns for micro gas analyzers. Anal. Chem. 81, 3471–3477 (2009). 19351142 10.1021/ac8027382

[b16] Radadia A. D., Salehi-Khojin A., Masel R. I. & Shannon M. A. The effect of microcolumn geometry on the performance of micro-gas chromatography columns for chip scale gas analyzers. Sens. Actuators B Chem. 150, 456–464 (2010).

[b17] Fan Y., Rubakhin S. S. & Sweedler J. V. Collection of peptides released from single neurons with particle-embedded monolithic capillaries followed by detection with matrix-assisted laser desorption/ionization mass spectrometry. Anal. Chem. 83, 9557–9563 (2011). 22053721 10.1021/ac202338e PMC3243447

[b18] Conn P. J. & Kaczmarek L. K. The bag cell neurons of Aplysia - a model for the study of the molecular mechanisms involved in the control of prolonged animal behaviors. Mol. Neurobiol. 3, 237–273 (1989). 2698177 10.1007/BF02740607

[b19] Hatcher N. G. & Sweedler J. V. Aplysia bag cells function as a distributed neurosecretory network. J. Neurophysiol. 99, 333–343 (2008). 18003877 10.1152/jn.00968.2007

[b20] Jakubowski J. A., Hatcher N. G. & Sweedler J. V. Online microdialysis-dynamic nanoelectrospray ionization-mass spectrometry for monitoring neuropeptide secretion. J. Mass Spectrom. 40, 924–931 (2005). 15934039 10.1002/jms.869

[b21] Smeal R. M., Rabbitt R., Biran R. & Tresco P. A. Substrate curvature influences the direction of nerve outgrowth. Ann. Biomed. Eng. 33, 376–382 (2005). 15868728 10.1007/s10439-005-1740-z

[b22] Smeal R. M. & Tresco P. A. The influence of substrate curvature on neurite outgrowth is cell type dependent. Exp. Neurol. 213, 281–292 (2008). 18602394 10.1016/j.expneurol.2008.05.026

[b23] Fukuda J. . Micropatterned cell co-cultures using layer-by-layer deposition of extracellular matrix components. Biomaterials 27, 1479–1486 (2006). 16242769 10.1016/j.biomaterials.2005.09.015

[b24] Charest J. L., Bryant L. E., Garcia A. J. & King W. P. Hot embossing for micropatterned cell substrates. Biomaterials 25, 4767–4775 (2004). 15120523 10.1016/j.biomaterials.2003.12.011

[b25] Angeli F., Brown G., Connolly P. & Uttamchandani D. Micromachined scaffolds as primers for cartilage cell growth. Micro Nano Lett. 1, 66–70 (2006).

[b26] Martinez D. . High-fidelity patch-clamp recordings from neurons cultured on a polymer microchip. Biomed. Microdevices 12, 977–985 (2010). 20694518 10.1007/s10544-010-9452-z

[b27] Zeck G. & Fromherz P. Noninvasive neuroelectronic interfacing with synaptically connected snail neurons immobilized on a semiconductor chip. Proc. Natl. Acad. Sci. USA 98, 10457–10462 (2001). 11526244 10.1073/pnas.181348698 PMC56982

[b28] Mahoney M. J., Chen R. R., Tan J. & Saltzman W. M. The influence of microchannels on neurite growth and architecture. Biomaterials 26, 771–778 (2005). 15350782 10.1016/j.biomaterials.2004.03.015

[b29] Metz S., Holzer R. & Renaud P. Polyimide-based microfluidic devices. Lab Chip 1, 29–34 (2001). 15100886 10.1039/b103896f

[b30] Yin N. F. . Microfluidic chip for peptide analysis with an integrated HPLC column, sample enrichment column, and nanoelectrospray tip. Anal. Chem. 77, 527–533 (2005). 15649049 10.1021/ac049068d

[b31] Lacour S. P. . Polyimide micro-channel arrays for peripheral nerve regenerative implants. Sens. Actuators A Phys. 147, 456–463 (2008).

[b32] Sun Y. . Assessment of the biocompatibility of photosensitive polyimide for implantable medical device use. J. Biomed. Mater. Res. 90A, 648–655 (2009). 10.1002/jbm.a.32125 18563817

[b33] Richardson R. R., Miller J. A. & Reichert W. M. Polyimides as biomaterials - preliminary biocompatibility testing. Biomaterials 14, 627–635 (1993). 8399958 10.1016/0142-9612(93)90183-3

[b34] Von Hoff D. D., Forseth B. J., Huong M., Buchok J. B. & Lathan B. Improved plating efficiencies for human-tumors cloned in capillary tubes versus Petri dishes. Cancer Res. 46, 4012–4017 (1986). 3731071

[b35] Sanford K. K., Earle W. R. & Likely G. D. The growth *in vitro* of single isolated tissue cells. J. Natl. Cancer Inst. 9, 229–246 (1948). 18105872

[b36] Garden R. W., Shippy S. A., Li L. J., Moroz T. P. & Sweedler J. V. Proteolytic processing of the Aplysia egg-laying hormone prohormone. Proc. Natl. Acad. Sci. USA 95, 3972–3977 (1998). 9520477 10.1073/pnas.95.7.3972 PMC19947

[b37] Ahmed W. W. . Mechanical tension modulates local and global vesicle dynamics in neurons. Cell. Mol. Bioeng. 5, 155–164 (2012). 23002399 10.1007/s12195-012-0223-1 PMC3445628

