# Supplementary material for: A neuron-in-capillary platform for facile collection and mass spectrometric characterization of a secreted neuropeptide
Source: Sci Rep. 2016 Jun 1;6:26940. doi: 10.1038/srep26940 (PMC4887886; doi:10.1038/srep26940)
Supplement: Supplementary Information [file srep26940-s1.doc]

**SUPPLEMENTARY INFORMATION**

A neuron-in-capillary platform for facile collection and mass spectrometric characterization of a secreted neuropeptide

Chang Young Lee,^a,b*^ Yi Fan,^a^ Stanislav S. Rubakhin,^a^ Sook Yoon,^b^ and Jonathan V. Sweedler^a*^

^a^ Department of Chemistry and the Beckman Institute, University of Illinois, Urbana, IL 61801, USA. Fax: 217 265 6290; Tel: 217 244 7359

^b^ School of Energy and Chemical Engineering, School of Life Sciences, Ulsan National Institute of Science and Technology (UNIST), Ulsan 44919, Republic of Korea. Fax: 82-52-217-2509; Tel: 82-52-217-2547

^*^ Corresponding authors: [*cylee@unist.ac.kr*](mailto:cylee@unist.ac.kr), and [*jsweedle@illinois.edu*](mailto:jsweedle@illinois.edu)

**
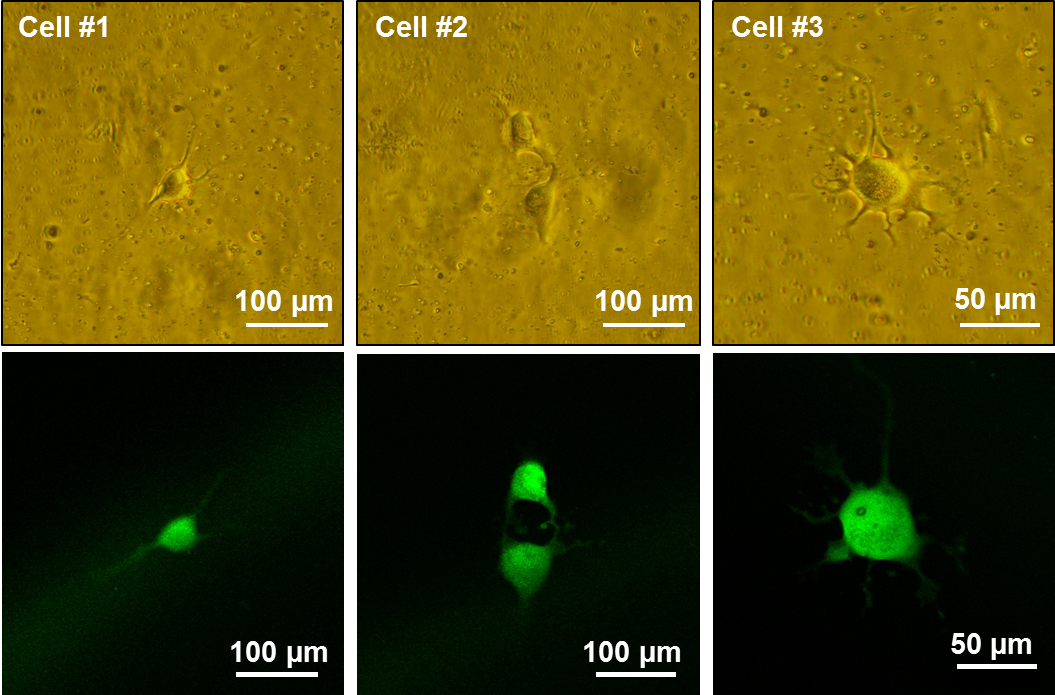
**

**Fig. S1.** Bag cell neurons of *Aplysia kurodai* cultured on a polyimide substrate and labeled with cell viability assay. Bag cell neurons were cultured on untreated Kapton® polyimide tape (1 days in vitro (DIV), top). Fluorescence images of the bag cells were captured in green channel using 488 nm laser excitation (1 DIV, bottom). Here the cells were stained with fluorescein diacetate (green), and confocal fluorescence images were acquired using Olympus FV1000 confocal microscope with a 20x objective. The results above confirms that the cell viability is not affected by polyimide.

**Fig. S2.** HEK293T cells cultured in a polyimide capillary. HEK293T cells (Korean Cell Line Bank, passages < 30) were cultured in a MEM (Gibco) supplemented with 10 % FBS, 50 units/mL penicillin, and 50 μg/mL streptomycin at 37 °C under 5 % CO_2_. Cells were were detached with Trypsin-EDTA (Gibco) and diluted into ~2×10^4^ cells/mL. Then the cell suspension was injected into the polyimide capillary. The capillary was incubated in a cell culture flask (100 pi) filled with media. Cells were then imaged after 3 days using EVOS Epi-fluorescence microscope (Life Technology).
